# Integrated Approach Including Docking, MD Simulations, and Network Analysis Highlights the Action Mechanism of the Cardiac hERG Activator RPR260243

**DOI:** 10.1021/acs.jcim.3c00596

**Published:** 2023-07-28

**Authors:** Flavio Costa, Riccardo Ocello, Carlo Guardiani, Alberto Giacomello, Matteo Masetti

**Affiliations:** † Dipartimento di Ingegneria Meccanica e Aerospaziale, Sapienza Università di Roma, via Eudossiana 18, 00184 Rome, Italy; ‡ Department of Pharmacy and Biotechnology, Alma Mater Studiorum−Università di Bologna, via Belmeloro 6, 40126 Bologna, Italy

## Abstract

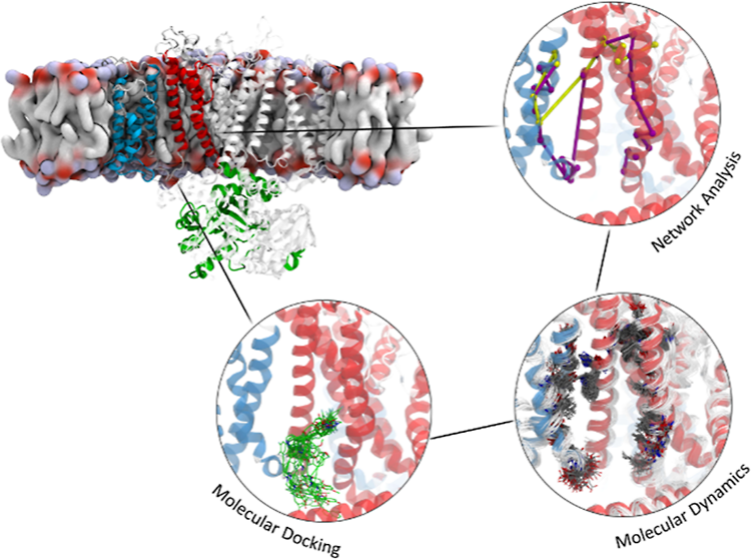

hERG is a voltage-gated potassium channel involved in the heart contraction whose defections are associated with the cardiac arrhythmia Long QT Syndrome type 2. The activator RPR260243 (RPR) represents a possible candidate to pharmacologically treat LQTS2 because it enhances the opening of the channel. However, the molecular detail of its action mechanism remains quite elusive. Here, we address the problem using a combination of docking, molecular dynamics simulations, and network analysis. We show that the drug preferably binds at the interface between the voltage sensor and the pore, enhancing the canonical activation path and determining a whole-structure rearrangement of the channel that slightly impairs inactivation.

## Introduction

**Figure 1:**
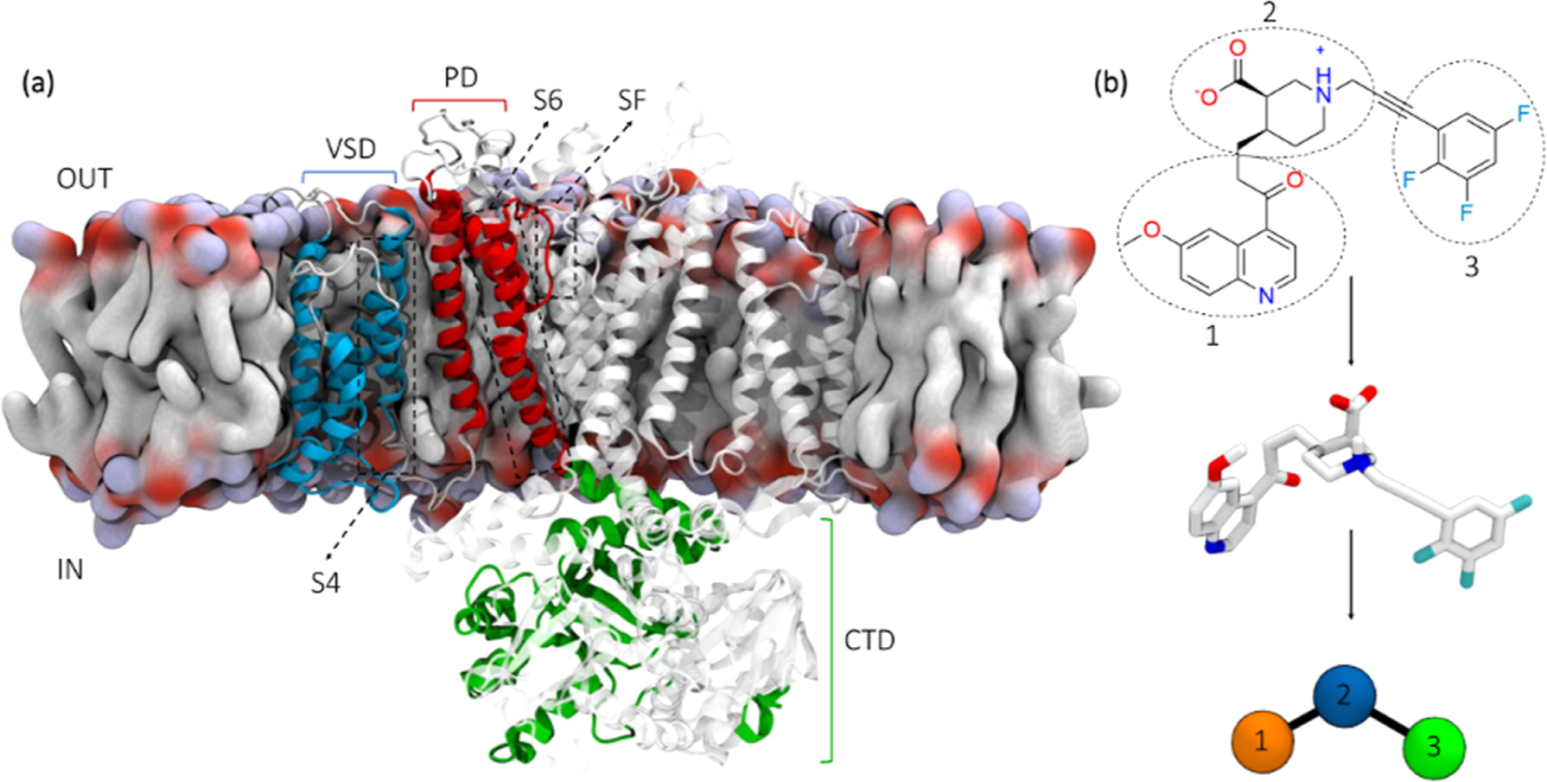
(a) Illustration of the hERG channel in the open state conformation embedded in a lipid membrane model. The VSD encompassing helices S1 to S4 is in blue, the PD with helices S5 and S6, the P-Loop, the SF is in red, and the carboxy-terminal domain is in green. (b) Structural formula of RPR and associated graph representation. Colored circles represent the network nodes corresponding to the center of mass of each numbered region.

The human Ether-à-go-go-related gene (hERG or *KCNH2*) codes a voltage-gated potassium channel expressed by heart cells^1^. It is involved in the delayed rectifier current (IK_r_), thus playing a key role in the repolarization phase of the cardiac action potential.^2^ Based on the protein architecture, hERG is classified as a non-domain-swapped channel where the voltage sensor domain (VSD), encompassing helices S1 to S4, contacts the pore domain [PD, i.e., helices S5 and S6, the P-Loop, and the selectivity filter (SF)] of the same subunit (Figure 1a).

hERG is characterized by three functional states: closed, open, and inactivated. Upon depolarization of the membrane potential, the motion of the S4 helix, carrying the gating charges, is transmitted to the PD where helices S6 that line the pore splay outwards, thus opening the channel.^3^ This motion follows two routes: a canonical path through the S4–S5 linker (L45) and S5 helix and a noncanonical path through helices S1 and S5.^4−6^ Then, the channel rapidly enters the inactivated state that has been suggested to be characterized by structural modifications of the SF which stop the ion’s flow^7,8^ although the molecular details of this process are not completely clarified. When the membrane slowly repolarizes, the channel rapidly recovers from inactivation and reopens thus ceasing the action potential. The closed-to-open transition is defined as “activation,” and the open-to-inactivated transition is defined as “inactivation.”

Long QT syndrome type 2 (LQTS2) is a pathology characterized by the prolongation of the QT interval in the electrocardiogram signal^9^ that promotes ventricular arrhythmia and sudden cardiac death.^3^ It can be induced by both genetic loss-of-function mutations on the hERG gene (congenital) or by the assumption of a wide range of unspecific drugs (acquired) including anti-arrhythmics, antibiotics, antihistamines, antidepressants, antimicrobials, anticancers, and antimalarials that blocking hERG^10^ determine a slower repolarization of the cardiac action potential thus causing LQTS2. To date, the main treatment is based on β-blockers that reduce the risk of fatal cardiac events.^11^ However, failure cases of this pharmacological therapy observed prevalently in children and women led to an alternative treatment with implantable cardioverter-defibrillator that, unfortunately, is very expensive.^12^ To reduce the access limit of effective therapies to patients, much interest has been raised in discovering and developing additional pharmacological strategies to treat LQTS2.

Molecules that increase the hERG activity by modulating channel gating are defined as “activators” and became of interest due to their potential use for reversing LQTS2. Several compounds have been identified as hERG activators that can be grouped into four classes depending on the action mechanism:^13^ class 1, slowed rate of channel deactivation; class 2, attenuation of inactivation; class 3, negative shift of the voltage dependence of activation; class 4, increase in channel open probability. However, in vivo tests showed that activators of class 2 and 3 have a proarrhythmic risk because they determine a too rapid repolarization of the cardiac action potential thus promoting short QT syndromes.^14^

**Figure 2:**
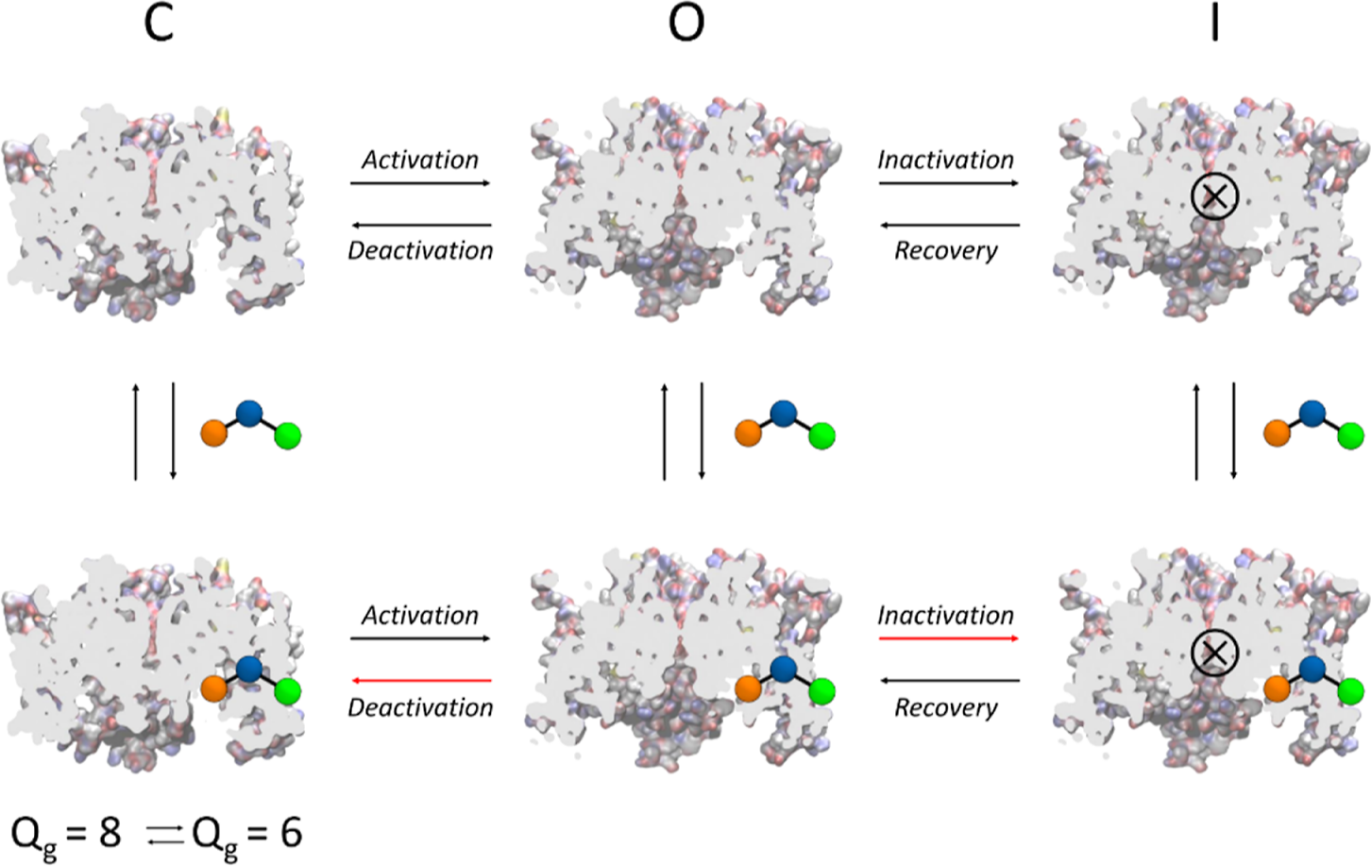
Scheme representing the action of RPR as a hERG activator. Red arrows refer to impaired transition from one state to another influenced by the drug. The closed states with gating charges *Q*_g_ = 8e and *Q*_g_ = 6e are assumed to be at equilibrium.

In this context, RPR260243 [(3*R*,4*R*)-4-[3-(6-methoxy-quinolin-4-yl)-3-oxo-propyl]-1-[3-(2,3,5-trifluoro-phenyl)-prop-2-ynyl]-piperidine-3-carboxylic acid], hereafter RPR (Figure 1b), is the first compound that has been designed to enhance hERG activity without excessive abbreviation of the cardiac action potential.^15^ It is an activator of class 1 because it significantly slows the rate of deactivation and slightly attenuates the inactivation (Figure 2).^16^ It has a high specificity to the isoform *b* of hERG, which lacks the entire long N-terminal domain.^17^ No activating effects were observed on other voltage-gated channels including the closely related ERG3 (K_V_11.3), a human brain potassium channel.^18^ Mutagenesis experiments of amino acids located in the loop L45 or cytoplasmic ends of the S5 and S6 helices showed that both the VSD and the PD play a role in interacting with RPR.^16^ This evidence suggests a possible binding site of RPR in or near the channel pore but the molecular determinants of the effects on the hERG gating are not clear and need to be further addressed.

In this work, we studied the effects of RPR on the hERG gating via a combination of docking, molecular dynamics (MD) simulations, and network analysis. First, from the equilibrium trajectories of the hERG open and closed unbound states, we identified the most representative conformations of the amino acids known to be involved in the interactions with RPR.^16^ Then, with ensemble docking calculations, the best poses of the drug inside the predicted binding sites were identified. Finally, after running equilibrium simulations on the open and closed states bound with RPR, a network analysis was adopted to microscopically describe the mechanism by which RPR influences the gating of the channel. Results show that the drug RPR enhances the canonical activation path. The present approach, which combines docking, MD simulations, and network analysis, shows promise to describe allostery in biological molecules, also in the presence of drugs.

## Methods

### MD Simulations

MD is rapidly emerging as a third pillar of science that complements theory and experiments both in material science^19,20^ and biophysics.^21−23^ MD simulations of the hERG unbound state were run for 500 ns from the open and closed states with gating charge *Q*_g_ = 8e and *Q*_g_ = 6e produced in Costa et al., 2022.^5^ Specifically, the open state was produced from the experimental structure (PDB ID 5VA2),^24^ while the closed state was generated through a combined approach based on homology modelling using such as a template EAG1 (PDB ID 5K7L)^25^ and Steered MD simulations to pull helix S4 in the closed position.^5^ The systems with gating charge *Q*_g_ = 8e and *Q*_g_ = 6e were used because the experimental value recorded by Zhang et al.,^26^ was 6.4e that is typically underestimated by 20% due to limits of the experimental technique.^27,28^ In the view of Mandala & MacKinnon 2022,^29^ the system with *Q*_g_ = 8e best represents the closed state of hERG (Figure S1) but, considering the uncertainty of the experimental charges and the potential relevance for intermediate states between the open and the closed ones, we reported both closed states (*Q*_g_ = 6e and *Q*_g_ = 8e). In both states, since the fragment connecting the PAS domain to the VSD was not experimentally resolved and it was too long to be predicted with state-of-the-art homology modelling tools, the N-terminal region was not included. Consequently, the simulated systems correspond to the hERG isoform *b* that lacks the entire PAS/Pas-cap domains.^30^

The bound systems were produced using the CHARMM-GUI membrane builder^31^ as follows: the bound open system #1 was embedded in bilayers of 915 1-palmitoyl-2-oleoyl-*sn*-glycero-3-phosphocholine lipids with 47,752 TIP3P water molecules and 0.15 M of KCl to form a simulation box of ca. 125 × 125 × 160 Å totaling 214,200 atoms; the bound open system #2 was embedded in bilayers of 903 1-palmitoyl-2-oleoyl-*sn*-glycero-3-phosphocholine lipids with 46,769 TIP3P water molecules and 0.15 M of KCl to form a simulation box of ca. 125 × 125 × 160 Å totaling 210,709 atoms; the bound closed system with *Q*_g_ = 6e was embedded in bilayers of 927 1-palmitoyl-2-oleoyl-*sn*-glycero-3-phosphocholine lipids with 49,673 TIP3P water molecules and 0.15 M of KCl to form a simulation box of ca. 125 × 125 × 160 Å totaling 220,509 atoms; the bound closed system with *Q*_g_ = 8e was embedded in bilayers of 921 1-palmitoyl-2-oleoyl-*sn*-glycero-3-phosphocholine lipids with 50,448 TIP3P water molecules and 0.15 M of KCl to form a simulation box of ca. 125 × 125 × 160 Å totaling 222,572 atoms. In each model only one drug was docked to the channel, thus having one RPR per simulation. The ligand was parametrized using the Antechamber tool included in CHARMM-GUI, adopting the General Amber Force Field (gaff2)^32^ and AM1-BCC method^33,34^ to calculate the ligand charges. The simulation protocol consisted in a first step of 150 ns restrained equilibration where all heavy atoms of the protein were restrained at their initial position with a force constant of 10 kcal/mol/Å^2^ followed by a 500 ns production run. Considering that RMSD reached a steady state during the first part of the simulations (see Figure S2), the last 300 ns of the production runs were used for collecting data. Three replicas per system were run, for a total of twelve independent simulations. The integrity of the conduction pore and of the SF was monitored during the simulations computing the radius profile with the HOLE program^35^ and the distances between G626 Cα of two opposite subunits as already done by Li et al., 2021 (Figure S3).^7^

All simulations were run with NAMD^36^ using the ff14SB force field for the protein,^37^ the Lipid17 force field for the lipids^38^ and the TIP3P water model.^39^ Pressure was kept at 1.01325 bar by the Nosé–Hoover Langevin piston method^40,41^ and the temperature was maintained at 303.15 K by a Langevin thermostat with a damping coefficient of 1 ps^–1^. Long-range electrostatic interactions were evaluated with the smooth PME algorithm^42^ with a grid space of 1 Å. For short-range non-bonded interactions, a cutoff of 12 Å with a switching function at 10.0 Å was used. The integration time step was 2 fs.

### Cluster Analysis

The representative structures (*n* = 10) of the conformations sampled during the hERG unbound state simulations were identified using the CPPTRAJ program of Amber package^43^ with the K-means clustering algorithm.^44^ A RMSD-based metric was used to evaluate the pairwise distance between conformations. Considering that Perry et al.,^16^ identified helices S5 and S6 to play a key role in the interactions with RPR, the following amino acids were considered for the cluster analysis: G546 to L559 on the S5 helix and S649 to Y667 on the S6 helix. The atomic equivalencies and the presence of four putative binding sites due to the homo-tetrameric architecture of the channel were explicitly considered during cluster analysis. The NanoShaper software^45^ was used to characterize the shape and size of the putative binding pockets for the representative structures of each cluster using default parameters.

### Ensemble Docking and Analysis of the Poses

Molecular docking calculations were performed using AutoDock-GPU(https://github.com/ccsb-scripps/AutoDock-GPU)^46,47^ on all the representative conformations extracted from the MD trajectories of the unbound state of the channel. The RPR activator was considered in the (3R,4R) absolute configuration. Among all the possible protonation states accessible for the ligand in solution, only the zwitterionic (hereafter referred to as Z) and neutral (N) forms were used in docking calculations. In particular, Z was considered because it represents the most abundant protonation state in solution at physiological pH. Instead, N represents the dominant form in a lipid environment, and it was included in the docking calculations because the putative binding site of RPR is expected to be localized near the membrane, and the possibility that the ligand might bind the channel in this form cannot be a priori ruled out. The piperidine ring was considered only in the chair conformation. Because of the absolute configuration of the two stereocenters, the carboxylic acid group in position 3 of the ring and the quinoline moiety of the molecule in position 4 (substituent R in Figure S4) are found in a syn relationship. As such, they always adopt opposite axial/equatorial orientations regardless of the pseudorotational state of the ring. Conversely, depending on the pyramidalization state of the piperidine nitrogen, the trifluoro-phenyl substituent in position 1 (substituent R1 in Figure S4) can be found either in the equatorial or axial orientation independently from the orientation of the groups in position 3 and 4. The combination of two stereocenters and the transient pseudo-stereocenter on the piperidine nitrogen resulted in a total of eight distinct states that should be considered as individual docking instances. Hereafter, we will refer to these states, and corresponding calculations, using a vector notation where the equatorial/axial orientation of the substituent is described by a binary attribute “a”/“e”, respectively, following the sequential position of the substituent found in the ring: 1, 3, and 4. Such a notation is appended to the “N”/“Z” prefix describing the protonation form of the molecule. The conformational states where R1 assumes an axial orientation were not considered, as they are expected to be much less favorable compared to the equatorial orientation. In summary, only four out of the eight possible states were explicitly considered for the docking calculation: Z(eea)—Lig. 1, Z(eae)—Lig. 2, N(eea)—Lig. 3, and N(eae)—Lig. 4 (see Figure S4).

The initial states of the ligand were generated using MarvinSketch 20.21.0 and then optimized using Gaussian16 C0.1^48^ at the B3LYP/6-31G* level of theory. Default parameters were used for the docking calculation, except for the grid spacing that was reduced to 0.250 Å with respect to the default value of 0.375 Å. A total number of 1000 poses were generated for each state of the ligand and for every channel conformation. The docking outcome was analyzed using the clustering algorithm implemented into AutoDock-GPU using an RMSD threshold of 2 Å, and the results were aggregated. Three scoring functions were used: (i) the original AutoDock^49^ scoring function (*E*_AD4_), (ii) a rescoring method considering the population of clustered poses (maximum probability, or “MaxP”), and (iii) a rescoring method considering both the population of the clustered poses and the relative population of the channel conformation (“MaxP’”). Only the lowest energy pose belonging to each cluster was considered. In other words, an energy , was assigned to each cluster, where *i* stands for the elements belonging to cluster *I*.

MaxP was defined as:^50,51^
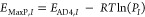
1where *P*_*I*_ represents the population of the *I*^th^-cluster of the ligand.

Similarly, the statistical weight of observing a given channel conformation was considered in MaxP′ as:
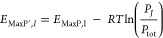
2where *P*_*J*_ is the population of the *J*^th^-cluster of the channel in which the docking calculation was carried out referred to as the total population of the ensemble.

### Network Analysis

To study the VSD-PD and VSD-SF coupling mechanisms, the hERG bound/unbound state was represented as a graph^52^ where nodes are the protein amino acids and edges the interactions between pairs. The activator RPR was split into three regions (as shown in Figure 1B) each corresponding to a node in the graph. The weight assigned to the edges was
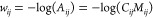
3where *C*_*ij*_ is a semi-binary contact map and *M*_*ij*_ is the mutual information matrix. Specifically, *C*_*ij*_ was computed with a truncated Gaussian kernel
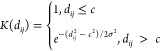
4where *d*_*ij*_ is the distance between the *C*_α_ of the *i* and *j* amino acids and *c* is the cutoff distance set to 7.0 Å. The width σ of the Gaussian kernel was chosen to attain a negligibly small value of the kernel at *d*_*ij*_ = 10Å. So, we imposed *K*(*d*_cut_) = 10^–5^ attaining σ = 1.48. The contact map was computed by averaging the value of the kernel over all the frames of the trajectory
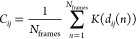
5

The mutual information *M*_*ij*_ of two random variables expresses their reciprocal independence or coupling and was used to quantify the motion correlation of two amino acids. Defining *d*_*i*_ and *d*_*j*_ as the displacement of the center of mass of the side chain with respect to its average position, the mutual information quantifies the loss of uncertainty on the position of amino acid *i* knowing the position of amino acid *j*
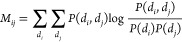
6

A normalized mutual information  was used where *H*_*ij*_ corresponds to the Shannon^53^ entropy of variables *d*_*i*_ and *d*_*j*_. Spheres of radius 7 Å were centered on key amino acids on helix S4 (R531), loop L45 (E544), SF (F627), and helix S6 (N658) to identify all amino acids inside them for at least 70% of the trajectory defining the source and sink regions. Minimal paths between source and sink regions were identified using Dijkstra’s algorithm^54^ where the corresponding length *d*_min_ represents the lowest value computed from eq 3. The role of each amino acid was quantified computing betweenness centrality calculation with Brandes’s algorithm^55^ as implemented in the NetworkX 3.0 library.^56^

## Results

The putative binding modes of the activator RPR were generated through molecular docking calculations using AutoDock-GPU.^46,47^ To consider target flexibility upon binding, the relaxed complex scheme was adopted.^57^ Specifically, the conformations of the target (i.e., the hERG channel) were first sampled through extensive MD simulations. Ensemble docking was then carried out on the 10 representative conformations of the channel obtained through cluster analysis performed on the MD trajectories. For the sake of clarity, we will refer to these representative conformations simply as “channel #”, where the # character stands for the number of the corresponding cluster. The structural stability of the “best” poses (as defined in the Methods section) was assessed through repeated MD simulations of the drug–target complex with three replicas each. Finally, the effect of RPR on the hERG gating was characterized by adopting a network analysis.

### Binding Pocket Identification and Analysis

Docking simulations require a preliminary knowledge of the binding pocket where the ligand must be accommodated. We defined the binding site of RPR by combining the experimental information from the scanning mutagenesis study by Perry et al.,^16^ with a visual inspection of our MD trajectories in the unbound state whose conduction pore integrity was conserved during the simulations (Figure S3). The purpose of this preliminary stage was to define the binding site in terms of a continuous set of residues located on helices S5 and S6. We selected a binding site comprising residues G546 to L559 on helix S5 and residues S649 to Y667 on S6. The residues identified in this preliminary stage underwent clustering analysis and the representative conformations of the 10 clusters attained for the open and the closed states of the channel were analyzed through the NanoShaper software^45^ to compute the enclosed volume of the binding pockets. For the open state, cluster #3 and cluster #7 displayed a pocket of remarkable size (1463.6 Å^3^) delimited by our selected residues that could reasonably host a quite large molecule like the activator considered in this study. For example, Figure S5 shows the three largest pockets identified in cluster #3. As shown in the inset, the pocket is near the set of residues identified by Perry et al.^16^ to influence the effect of RPR on hERG gating. Interestingly, the pocket adopts a Y-shaped conformation with a deeper branch extending between S4 and S5 and a shallower one between S5 and S6. Notably, in cluster #7, these two sub-pockets appeared to be almost comparable in size. Conversely, in the closed states, the identification of a well-defined and broad pocket was not as satisfactory as in the open state, suggesting a more superficial interaction of the drug with the channel.

### Characterization of the Putative Binding Modes

Once the putative binding site was identified, ensemble docking calculations were performed considering different protein conformations for all the channel states. Moreover, for every channel conformation, four docking instances were carried out to describe the activator in the following configurations (see Methods for details): Z(eea)—Lig. 1, Z(eae)—Lig. 2, N(eea)—Lig. 3, and N(eae)—Lig. 4. The results were aggregated for all the conformational and protonation states of the ligand, and the rescoring methods previously described were applied to every conformation of the channel and of the ligand. The 10 top-ranking solutions obtained using the MaxP and MaxP′ rescoring functions were visually inspected to judge their reliability in terms of chemical intuition. We note that ranking the binding modes of multiple states of the ligand solely based on their energy is not formally correct as we are implicitly assuming the same stability in the unbound state. However, a high degree of consistency observed between binding modes of the activator considered in distinct states for the same channel conformation makes us confident that this approximation can be acceptable for the considered case.

**Figure 3:**
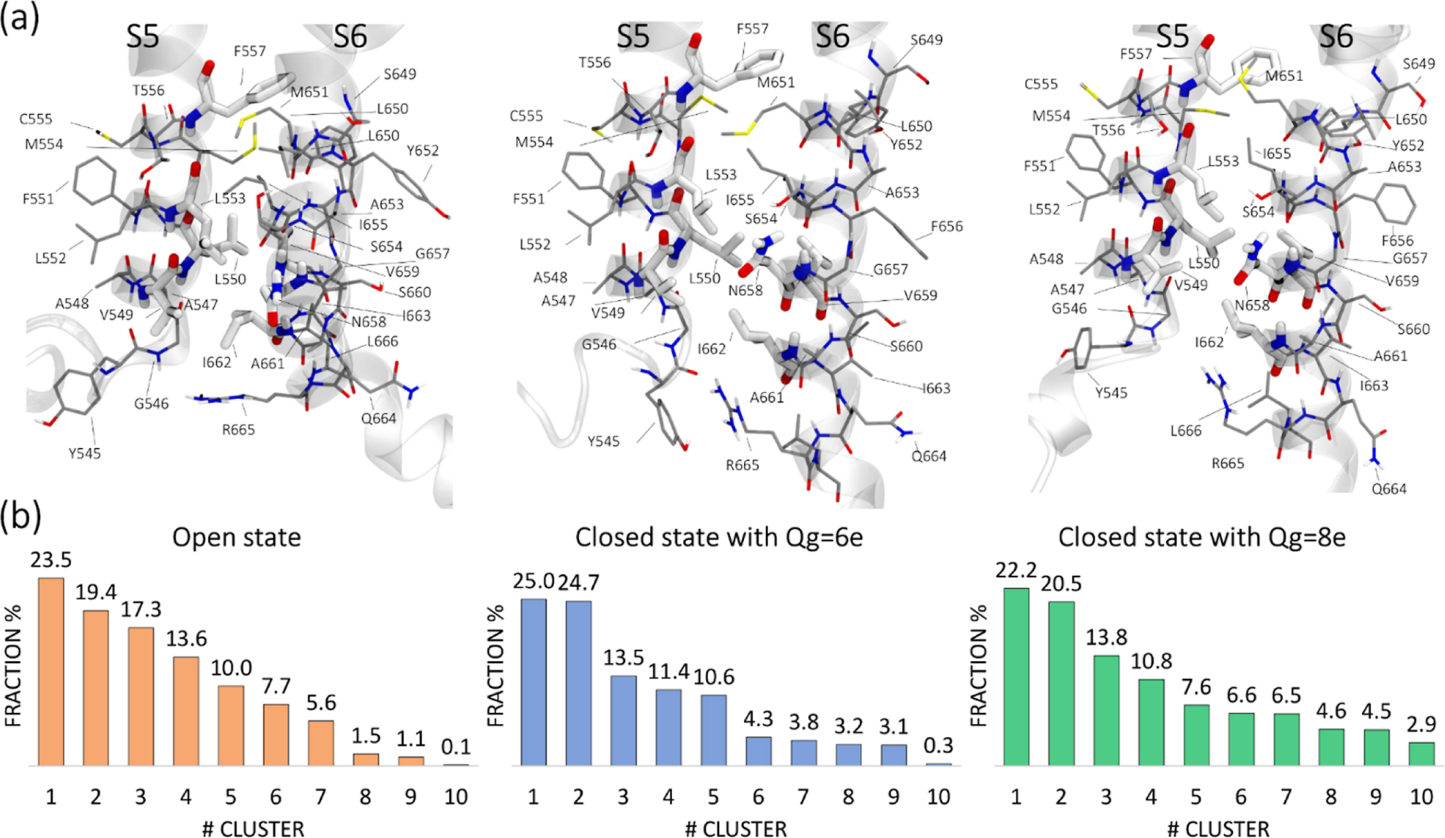
(a) Residues employed in the cluster analysis with respect to those directly implied in the activity of RPR displayed as thick capped sticks. (b) Relative population of the ten clusters identified for each channel model. The extended set of residues shown in panel (a) refers to the representative conformation of the most populated cluster (cluster #1) for each channel model.

In the open state, according to the MaxP rescoring method, the top-ranked docking solution consisted in Lig. 2 bound to channel 7. This specific binding mode, featuring the activator in the zwitterionic state, resulted in the best compromise between the bare AutoDock score and the population of the clustered open channel configurations. Notably, the relative population of cluster #7 was only 5.6% (see Figure 3). By applying the MaxP′ rescoring method that includes a penalty for infrequently sampled channel conformations, the previously described binding mode moved to rank 6 whereas the most stable binding mode became that where the ligand bound to channel 3 (relative population of 17.3%, Figure 3). Not surprisingly, channels 3 and 7 displayed a better-shaped binding pocket near the residues known to be directly implied in the activity of RPR, as identified by NanoShaper.^45^ Conversely, in both the closed states with different gating charges, a consensus was obtained between the binding mode prioritized through the employed rescoring methods. This occurrence reflects the fact that the best compromise between the AutoDock score, and the population of the clustered poses was reached among the most visited conformations of the channel when considered in the closed state. Specifically, the model with *Q*_g_ = 6e showed in top-rank position Lig. 2 bound to channel 3 (relative population of 13.5%), whereas the neutral state of the ligand (Lig. 3) bound to channel 2 (relative population of 20.5%) was preferred in the case of the model with *Q*_g_ = 8e.

**Figure 4:**
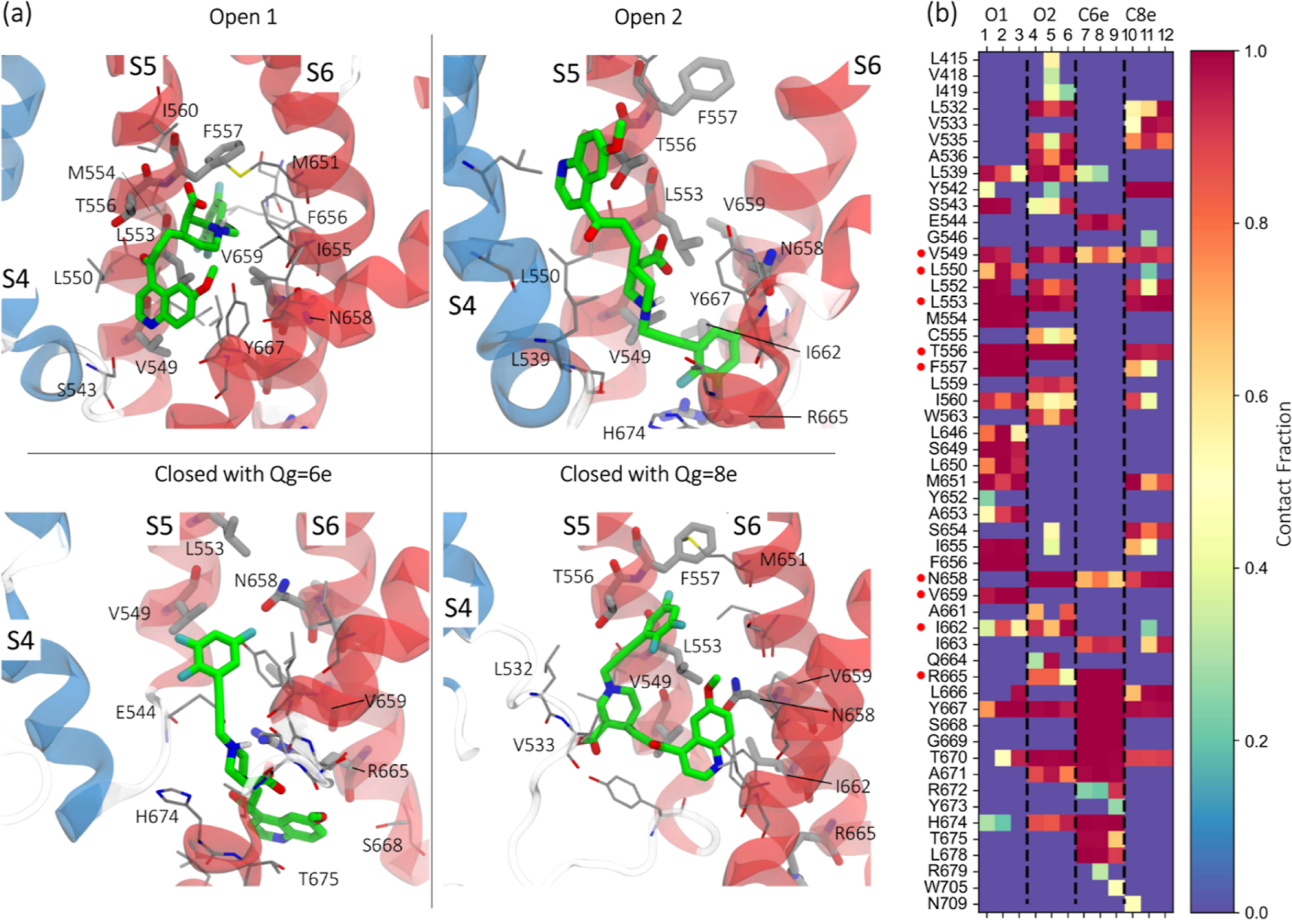
(a) Binding modes obtained through ensemble docking calculation where only residues 4.5 Å from the ligand are reported. (b) Contact map related to the ensemble docking simulations in (a) showing the presence of the interactions between RPR and residues that form the predicted binding sites during the overall simulations of the bound systems. A contact fraction equal to 1 indicates that the interaction between the ligand and the residue persists during the whole trajectory. The cutoff distance used to identify the interactions between the ligand and the side chain of residues was 4.5 Å. Residues known to affect the RPR activity are shown as sticks in panel (a) and are highlighted with red circles in panel (b).

The use of two rescoring methods combined with the systematic enumeration of channel conformations and ligand states allowed us to objectively identify the binding poses (Figure 4a) that have been considered for the next network analysis. In the following, the bound complexes of the open state hERG will be referred to as Open1 and Open2 for the best solution of Lig. 2 in channels 7 and 3 according to the MaxP and MaxP′ rescoring methods, respectively. The pose adopted by the drug in these states can be considered as an alternative binding mode of the activator where the main difference was a rotation of the major molecular axis along the membrane normal. Indeed, while in the case of Open2 the trifluoro-phenyl substituent in position 1 of the piperazine ring was directed towards the intracellular side of the membrane, in Open1 the innermost regions of the channel were reached through the quinoline moiety of the molecule. Specifically, in the former binding mode, the trifluoro-phenyl substituent engaged I662 and R665 through van der Waals and electrostatic interactions, respectively, whereas a cluster of hydrophobic residues including V549, L550, L553, and Y667, among the others, was involved in the interaction with the quinoline group in the latter. In both cases, the zwitterionic group in the central six-membered ring of the molecule did not seem to establish specific electrostatic interactions with protein residues. In the case of Open1, the trifluoro-phenyl substituent was deeply buried in a hydrophobic cleft of the binding site located between S5 and S6, whereas the shallower branch of the pocket (S4–S5) was contacted by the quinoline group in the case of Open2. The binding modes obtained for RPR in the two closed states were translated toward a lower region of the channel near the intracellular side of the membrane. This depends on the presence of a less structured binding pocket than that in the open state which forces the activator to optimize the interactions with the available residues on the surface of the channel. In both cases, the activator was shown to point the trifluoro-phenyl group toward the extracellular side of the membrane, similar to Open1.

MD simulations of the hERG-bound states showed that the prioritized binding modes were thermally stable (Figure S6). Moreover, the contact analysis (Figure 4b) confirmed the docking outcomes. Specifically, by comparing the two binding modes obtained for the open states, it was possible to notice a greater prevalence of highly preserved contacts in Open1 such as L550, L553, T556, F557, V659 that have been identified by Perry et al.,^16^ and M554, I560, L646, S649, L650, M651, A653, I655, F656, I662, Y667. Most of these residues are hydrophobic so the trifluoro-phenyl substituent remained firmly bound to the deepest branch of the binding pocket (S5 and S6 helices) over the simulations. The binding mode displayed by Open2 was also stable, but the analysis showed a more scattered preservation of contacts which was associated with the presence of shallower branches of the binding pocket. Perhaps, the most interesting features of Open2, highlighted by the contact analysis, was the presence of a charge-assisted hydrogen bond between the carboxylic acid group in position 3 of the piperazine ring and N658 as well as the hydroxyl group of T556, which are both important for RPR activity and that were not contacted in Open1. Interestingly, the interaction with N658 has been recently reported in the binding mode of RPR shown in the work by Zangerl-Plessl et al.,^58^ even though its orientation seems to be similar to that of Open1. In the closed states, the contact analysis showed a distinctive behavior for RPR bound to the closed model with *Q*_g_ = 6e, consistently with a significant slide of the whole molecule towards the intracellular side of the membrane. The analysis revealed that only two residues, N658 and R665, known to be involved in the interactions with RPR,^16^ formed contacts with the drug. Conversely, the binding mode obtained in the closed system with *Q*_g_ = 8e showed a contact pattern that closely resembled that obtained with Open2. Considering the contact similarity and the orientation of the molecule, these poses could be considered as the same binding mode captured in two different moments of the activation and deactivation processes.

### Effects of RPR on the hERG Allosteric Gating Paths

A network analysis was adopted to reveal the microscopic effects of RPR on the hERG gating where the protein was represented as a graph^52^ with nodes corresponding to the amino acids. In the spirit of Westerlund et al.,^59^ the activator was included in the network. It was split into three regions where the associated center of mass corresponds to a single node (Figure 1b). Finally, a weight expressed as *w*_*ij*_ = −log(*C*_*ij*_*M*_*ij*_) was assigned to each edge to quantify the allosteric path in terms of contacts and motions. The shortest paths between the VSD and the PD were determined via Dijkstra’s algorithm.^54^ According to previous works,^5,6,60^ the activation and deactivation paths were predicted on the closed states. This choice relies on the experimental evidence that the open state is more stable than the closed state thus, without an electric field, the channel spontaneously reaches the open state.^61−66^ This evidence suggests that the communication paths for activation and deactivation are not present in the open state, and they gradually build as the channel approaches the closed state. In this context, we assumed that the activation and deactivation paths follow the same route in both the open-to-closed and closed-to-open transitions. On the other hand, the inactivation paths were predicted on the open state because no structure has been experimentally solved for the inactivated state that is supposed to differ from that in the open state only for little modifications at the level of the SF.^7,24^ It is important to stress that the network analysis was run on equilibrium trajectories. However, as in previous works,^60,67,68^ it can be assumed that the paths discussed in the next paragraphs represent the initial motions occurring in the channel during the activation and the inactivation. The integrity of the conduction pore was preserved during the whole simulation time in both the unbound and the bound systems (Figure S3).

**Figure 5:**
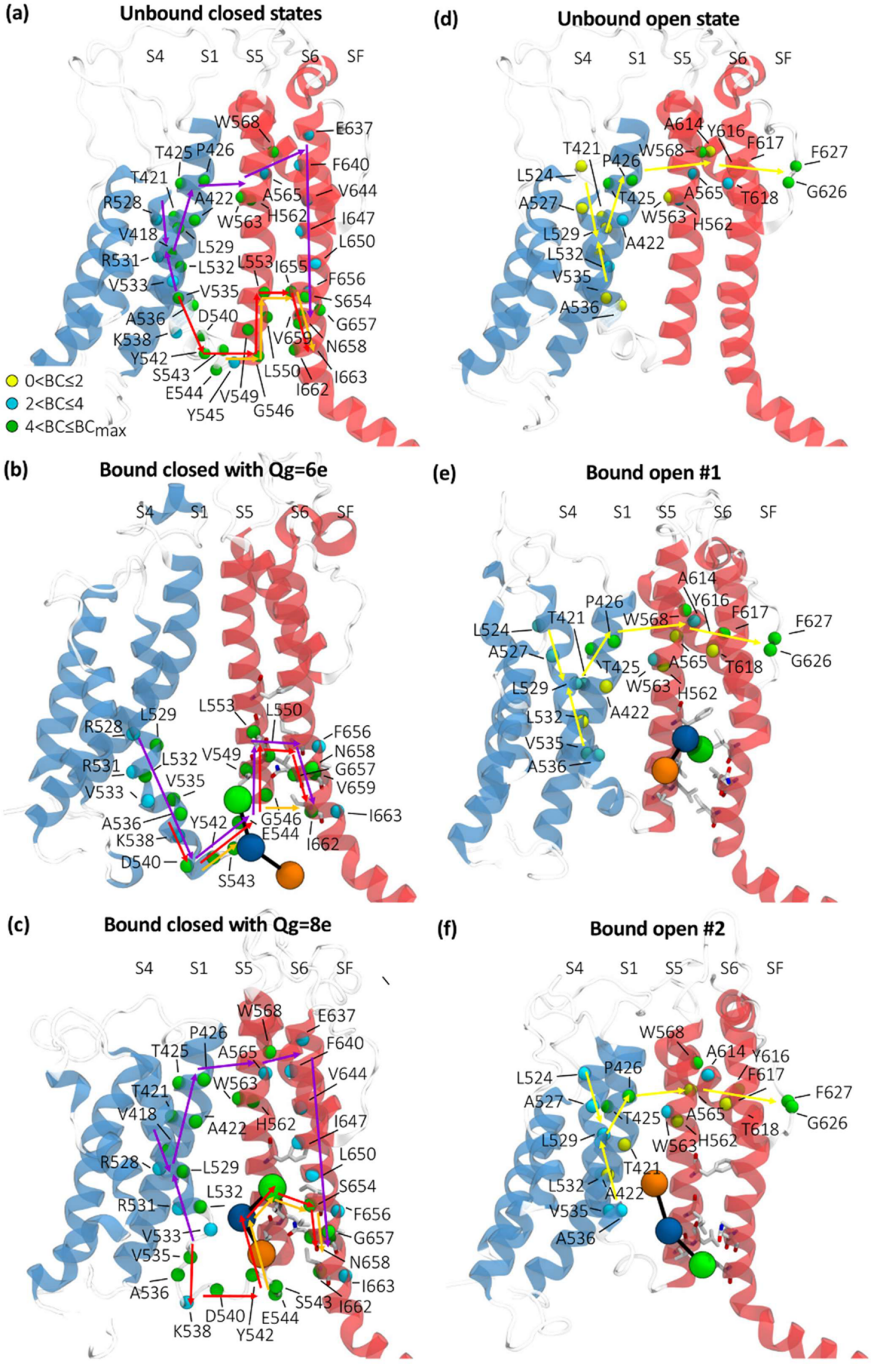
Activation and inactivation paths identified by the network analysis in the hERG closed and open states. Activation paths identified in the unbound (a) and bound (b,c) closed states with *Q*_g_ = 6e and *Q*_g_ = 8e, respectively. Inactivation path identified in the unbound (d) and bound (e,f) open states. RPR is schematized as described in Figure 1b. Colored arrows describe the kinematic chain of amino acids: violet arrows refer to the noncanonical activation path that follows the S4 → S1 → S5 → S6 route; red and orange arrows refer to the canonical paths L45 → S5 → S6 starting from the N-term or C-term sides of the loop, respectively; yellow arrows refer to the inactivation path that follows the S4 → S1 → S5 → P-Loop → SF route. Residues are colored based on the Betweenness Centrality values whose legend is indicated in the figure.

In the hERG unbound closed states, two families of activation paths were identified, as already described in Costa et al., 2022:^5^ the canonical path passing through the loop L45 and helix S5 divided into two subgroups based on the side of origin with respect to the loop L45 (red and orange arrows in Figure 5a, respectively); the noncanonical path that involves helices S1 and S5 (violet arrows in Figure 5a). When the activator RPR was docked at the C-terminal side of helix S6 of the system with *Q*_g_ = 6e as schematized in Figure 5b, the activation paths of that subunit were modified. Indeed, the canonical path from the N-terminal side of the loop L45 remained qualitatively the same but with a lower length *d*_min_, from *d*_min_ ∼ 17 in the unbound state to *d*_min_ ∼ 13 in the bound state as reported in Table S1 (red arrows in Figure 5b). The main effects were observed for the path originating from the C-terminal side of the loop that became extremely shorter than that in the hERG unbound state (from *d*_min_ ∼ 13 in the unbound state to *d*_min_ ∼ 4 in the bound state; orange arrows in Figure 5b). Shorter paths mean stronger information transfer and, consequently, enhanced activation. Considering the logarithmic nature of the metric used in the network analysis, a difference of nine units in *d*_min_ (e.g., the canonical activation path originating from the C-terminal side of the loop L45) corresponds to a difference of 4 orders of magnitude in terms of coupling efficiency suggesting that in the hERG bound subunit the VSD-PD coupling is extremely improved. Moreover, the paths originating from the upper part of helix S4 instead of following the noncanonical route were diverted to the canonical one. In short sum, the noncanonical path disappeared and the canonical one was reinforced. The VSD-PD coupling path became stronger in terms of information transfer, mostly due to the paths starting from the C-terminal side of the loop L45, confirming the functional asymmetry between the ends of loop L45 previously reported in experimental works on the hERG split channels.^69^

In a similar way to *Q*_g_ = 6e, when RPR was docked at the interface of helices S5 and S6 in the system with *Q*_g_ = 8e, the canonical activation paths changed leaving helix S5 and passing through the drug that acts as a bridge between the loop L45 and helix S6 (Figure 5c). Although the presence of the drug improved the VSD-to-PD information transfer (i.e., shorter path lengths *d*_min_ than in the unbound state), this improvement was less striking than that observed for the system with *Q*_g_ = 6e. For *Q*_g_ = 8e, the noncanonical activation path was not modified by the presence of RPR. Interestingly, in both the closed systems, the subunits without RPR showed a canonical activation path starting from the N-terminal side of the loop L45 shorter by three units than those of the unbound state (Table S1). This evidence suggests a possible cooperative effect of the activator on all the subunits.

The network analysis of the hERG unbound open state revealed an inactivation path (yellow arrows in Figure 5d) that involves the S4/S1 and S1/S5 subunit interfaces, as previously described in Bassetto et al.^6^ No effects were detected when the drug was positioned between helices S5 and S6 (Figure 5e). When RPR was docked between the loop L45 and the helices S1 and S5 (Figure 5f) *d*_min_ was slightly increased, meaning that the inactivation mechanism is slightly impaired. Considering the effects of RPR induced on the paths (i.e., a slight impairment of inactivation in the open bound system #2), it can be hypothesized that the binding site of the activator is localized at the VSD-PD interface instead of between helices S5 and S6.

## Discussion and Conclusions

LQTS2 is a cardiac pathology caused by genetic or acquired defections of the hERG channels that may lead to sudden death.^3^ To increase the accessibility to therapies, much interest has been raised in discovering new pharmacological strategies to treat LQTS2. In this context, one of the best candidates is RPR, an activator selective only to hERG that does not induce negative effects on the cardiac action potential.^15^ Previous experimental works revealed that mutations of residues located near the cytoplasmic ends of helices S5 and S6 influence the effects of RPR on the gating of the channel defining a putative binding site for the activator.^16^ Here, using a computational approach based on ensemble docking and network analysis, we described the action mechanism of RPR clarifying the molecular determinants of its interference with the electromechanical VSD-PD gating coupling.

As of the writing of this paper, we became aware of a work by Zangerl-Plessl et al.,^58^ on the RPR-hERG interaction where the electrophysiological analysis shows that the activation efficacy of RPR was not impaired by the removal of the N-terminal domain. This provides strong experimental support to our work where the PAS domain of the hERG channel was not included in the structure. Even if the authors applied ensemble docking like in the present case, the binding site they identified is deeply buried between helices S5 and S6. In the present work, we show that the binding pocket is Y-shaped with a deeper branch between S4 and S5 and a shallower one between S5 and S6. This disagreement is possible since we also analyzed the closed-state models. To assess this discrepancy that depends on the channel state used in the docking calculations, further experiments are suggested to clarify which state the drug preferably binds, also including mutations on the VSD.

The network analysis reveals two mechanisms of action of RPR. Firstly, a direct mechanism was observed in the closed system with gating charge *Q*_g_ = 8e where the drug favors the VSD-PD coupling acting as a bridge between the loop L45 and helix S6. Similarly, in the closed system with gating charge *Q*_g_ = 6e, the canonical path was strongly potentiated by the drug that strengthened the communication between the VSD and the PD pushing helix S6 towards helix S5. Interestingly, regardless of the gating charge, for both the closed states, the paths originating from the C-terminal side of the loop L45 were greatly modified by the drug. These effects depend on the stabilization of the conformation of the loop L45 at the S5-side determined by the drug that, in this way, enhances the ability of the loop to interact with helix S6. This evidence agrees with previous computational^5^ and experimental^69^ works in which loop L45 showed an asymmetrical behavior to open the channel where a cut at the C-terminal side of the loop would not impair the ability of the channel to open and close.

An indirect effect was also observed in the closed state with gating charge *Q*_g_ = 6e. The noncanonical activation path was diverted to the canonical one passing through the loop L45 and helix S5. In this case, the presence of the drug influenced the structure of the whole subunit, not only at the level of the PD, resulting in a loss of contacts between amino acids of helices S4–S1 and S1–S5 that interrupts the original noncanonical activation path. However, the overall effect was an enhanced activation because this new path has a shorter length than that of the noncanonical one which it replaces (Table S1). In the same way, when RPR was docked at the VSD-PD subunit interface in the open state, the inactivation was slightly impaired. Since the inactivation path remained qualitatively the same, this effect does not depend on modifications of the contact pattern but on less correlated motions of amino acids on helices S1 and S5.

Considering that the major effects of the drug were detected in the closed system with *Q*_g_ = 6e and in the open system with the #1 pose, we suggest that the binding site of RPR is located at the VSD-PD interface. Indeed, most of the residues V549, L550, L553, F557, N658, V659, I662, and L666 know to alter the effects of the drug if mutated to Ala^16^ interact with the drug (Figure 4b). Moreover, we showed that the presence of RPR in the closed systems influences the canonical activation paths originating from the N-terminal side of the loop L45 not only of the subunit with the drug but also in all the others. This evidence underlies a cooperative mechanism of the activator that could explain the previously shown dose-dependent effects of the drug on the hERG gating,^70^ to be quantitatively assessed in future work. As a final note, we wish to highlight that the direct involvement of the above-mentioned residues in drug binding is not necessarily implied by the bare mutagenesis data, as residues affecting the communication can also be located in the middle of the pathways. However, while we cannot rule out the presence of alternative or additional binding sites for the activator, our network analysis strongly supports the picture of an allosteric mechanism triggered by RPR binding at the VSD-PD interface.

To sum up, our results provided microscopic insights on the action mechanism of the activator RPR on the gating of the hERG channel. Moreover, the identification of its binding site could provide the basis for a rational design of drugs to optimize better candidates to treat LQTS2.

## Supporting Information Available

Minimal path lengths of the allosteric paths; comparison between the closed configuration of the VSD of hERG closed state with *Q*_g_ = 8e and of EAG; RMSD profiles of the hERG bound/unbound states; radius profiles and distances between alpha carbons of G626 in all the systems; scheme of the RPR structure; representation of the pockets of the channel #3 in the open state; RMSD profiles of the ligand inside the predicted binding sites (PDF)

## Notes

The MD trajectories, an example of the input file, and the topology files are openly available in ZENODO at https://doi.org/10.5281/zenodo.7840207.
