# Supplementary material for: Integrated Approach Including Docking, MD Simulations, and Network Analysis Highlights the Action Mechanism of the Cardiac hERG Activator RPR260243
Source: J Chem Inf Model. 2023 Jul 28;63(15):4888–99. doi: 10.1021/acs.jcim.3c00596 (PMC10428221; doi:10.1021/acs.jcim.3c00596)
Supplement: Supplementary file 1 — ci3c00596_si_001.pdf [file ci3c00596_si_001.pdf]

SUPPORTING INFORMATION

Integrated Approach Including Docking, MD Simulations, and Network Analysis Highlights the Action Mechanism of the Cardiac hERG Activator RPR260243

Flavio Costa [1†], Riccardo Ocello [2†], Carlo Guardiani [1], Alberto Giacomello [1] and Matteo Masetti [2*]*

[1] Dipartimento di Ingegneria Meccanica e Aerospaziale, Sapienza Università di Roma, via Eudossiana 18, 00184 Rome, Italy

[2] Department of Pharmacy and Biotechnology, Alma Mater Studiorum−Università di Bologna, via Belmeloro 6, 40126 Bologna, Italy

[†] These authors contributed equally

Corresponding authors emails: alberto.giacomello@uniroma1.it; matteo.masetti4@unibo.it

Table S1*. Minimal path lengths of allosteric paths identified by the network analysis for each subunit in the bound/unbound open and closed states. Blue values refer to the subunit with RPR.*

	**Unbound C Qg=6e**	**Unbound C Qg=8e**	**Bound C Qg=6**			**Bound C Qg=8e**			**Unbound O**	**Bound O #1**			**Bound O #2**		
			**#1**	**#2**	**#3**	**#1**	**#2**	**#3**		**#1**	**#2**	**#3**	**#1**	**#2**	**#3**
**L45 N-Term (I) → S6 (I)**	15,94	15,00	14,70	12,63	12,07	13,79	12,70	11,43	23,32	20,24	22,54	20,93	22,94	23,63	20,68
**L45 C-Term (I) → S6 (I)**	11,07	12,30	9,50	7,91	9,61	9,75	12,31	8,58	17,67	16,17	18,14	16,47	17,39	18,41	18,35
**L45 (IV) → S6 (I)**	10,26	21,00	11,49	11,30	11,89	19,38	16,32	16,41	25,48	22,38	25,23	21,48	24,65	22,74	22,16
**S4 (I) → S6 (I)**	17,74	16,70	15,70	14,34	13,64	17,11	15,85	13,10	18,97	18,91	20,75	19,10	19,29	20,23	19,04
**S4 (I) → SF (I)**	26,23	25,64	26,30	26,25	26,97	25,13	26,53	26,00	18,63	21,10	21,82	22,44	17,16	19,47	19,83
**L45 N-Term (II) → S6 (II)**	18,29	12,97	10,92	13,16	13,60	11,37	13,62	10,42	18,69	20,45	21,43	20,27	18,68	21,22	19,41
**L45 C-Term (II) → S6 (II)**	13,84	8,89	12,72	11,95	12,19	8,52	10,49	5,23	17,48	14,74	15,20	16,48	14,08	18,76	16,14
**L45 (I) → S6 (II)**	16,64	19,67	15,41	14,04	15,71	15,64	14,98	13,82	26,17	24,42	25,28	24,22	24,97	25,52	23,18
**S4 (II) → S6 (II)**	18,94	17,40	15,03	15,60	15,60	16,94	16,99	14,18	18,92	19,22	19,91	19,89	17,34	18,90	15,72
**S4 (II) → SF (II)**	26,08	25,96	26,41	26,61	26,76	25,79	25,62	25,29	19,54	21,16	22,43	22,18	20,12	20,96	18,70
**L45 N-Term (III) → S6 (III)**	17,37	15,47	12,73	13,90	13,08	13,44	12,08	9,32	24,82	20,02	22,19	25,44	25,06	24,18	23,22
**L45 C-Term (III) → S6 (III)**	13,72	14,15	15,23	13,21	12,84	12,48	9,74	7,07	20,03	14,41	17,78	19,75	20,78	18,92	19,26
**L45 (II) → S6 (III)**	18,78	20,24	16,00	17,57	19,33	17,90	17,05	12,09	24,61	22,06	26,71	24,12	23,13	23,90	22,30
**S4 (III) → S6 (III)**	17,80	14,07	17,25	17,07	17,64	14,58	14,33	10,40	19,00	18,10	20,50	22,05	22,04	21,75	19,81
**S4 (III) → SF (III)**	26,28	25,81	26,89	26,90	26,08	25,81	25,68	25,64	20,94	18,35	22,26	22,34	19,65	21,38	16,84
**L45 N-Term (IV) → S6 (IV)**	15,94	15,22	12,50	12,21	13,79	12,13	10,79	11,37	23,02	23,35	20,82	20,76	20,80	20,24	21,15
**L45 C-Term (IV) → S6 (IV)**	12,83	12,55	3,80	4,03	4,16	14,08	10,59	8,53	17,78	18,77	20,21	16,83	18,61	18,08	19,19
**L45 (III) → S6 (IV)**	15,72	19,55	18,12	18,14	17,55	18,73	18,26	13,50	23,02	22,84	22,23	23,16	25,37	23,96	22,87
**S4 (IV) → S6 (IV)**	17,24	16,07	14,93	14,00	15,51	15,30	12,27	13,35	18,98	21,95	18,86	19,97	18,79	18,73	21,17
**S4 (IV) → SF (IV)**	26,96	25,81	26,05	26,29	26,34	25,61	25,18	25,56	17,96	20,37	19,41	21,57	17,94	19,34	20,85

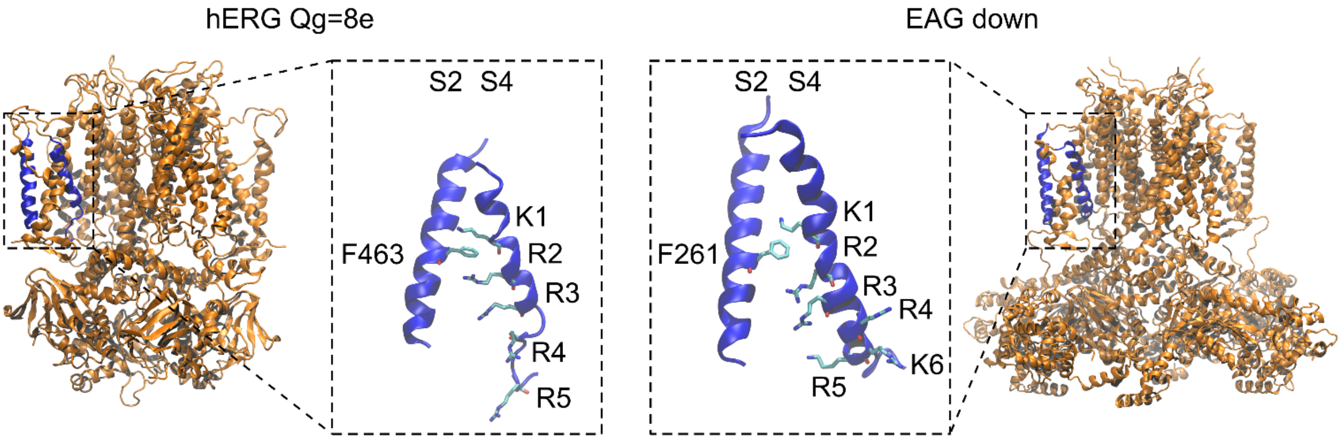

Figure S1. *Comparison between the down configuration of the VSDs in our predicted hERG closed state with* Q_g*=8e and in EAG* K_v *channel (PDB ID: 8EP1).*

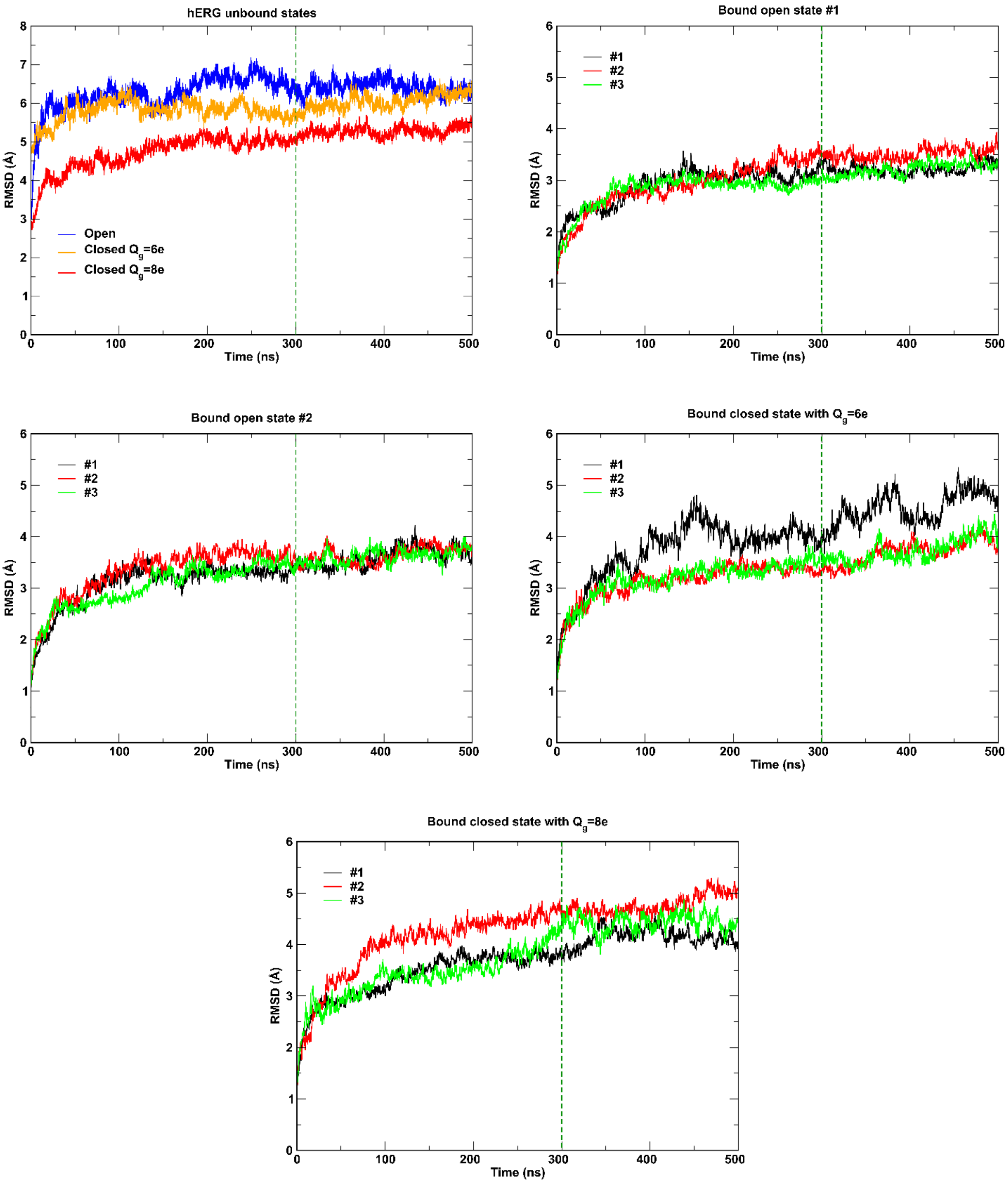

Figure S2. *RMSD profiles of the hERG bound/unbound states computed for the backbone of the protein. The reference conformations correspond to the structure of the channel at the beginning of the production phase in MD simulations.*

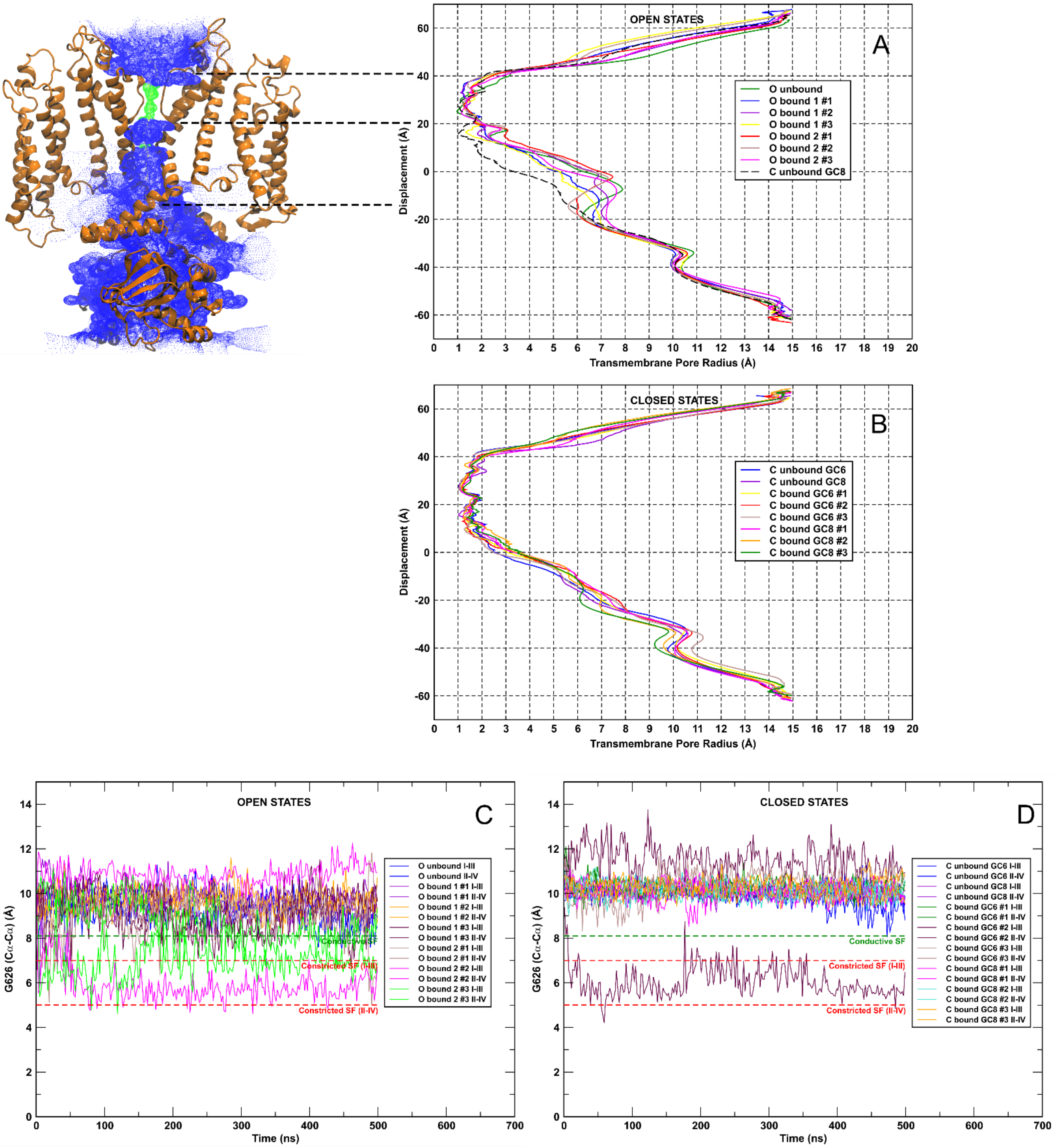

Figure S3. *Pore radius profiles in the open (A) and closed (B) states. Profile distances between G626 Cα of two opposite subunits in the open (C) and closed (D) states. In panel C and D, the dotted lines refer to the G626 Cα distance values indicated by Li et al, 2021 for the conductive (green) and constricted SF (red).*

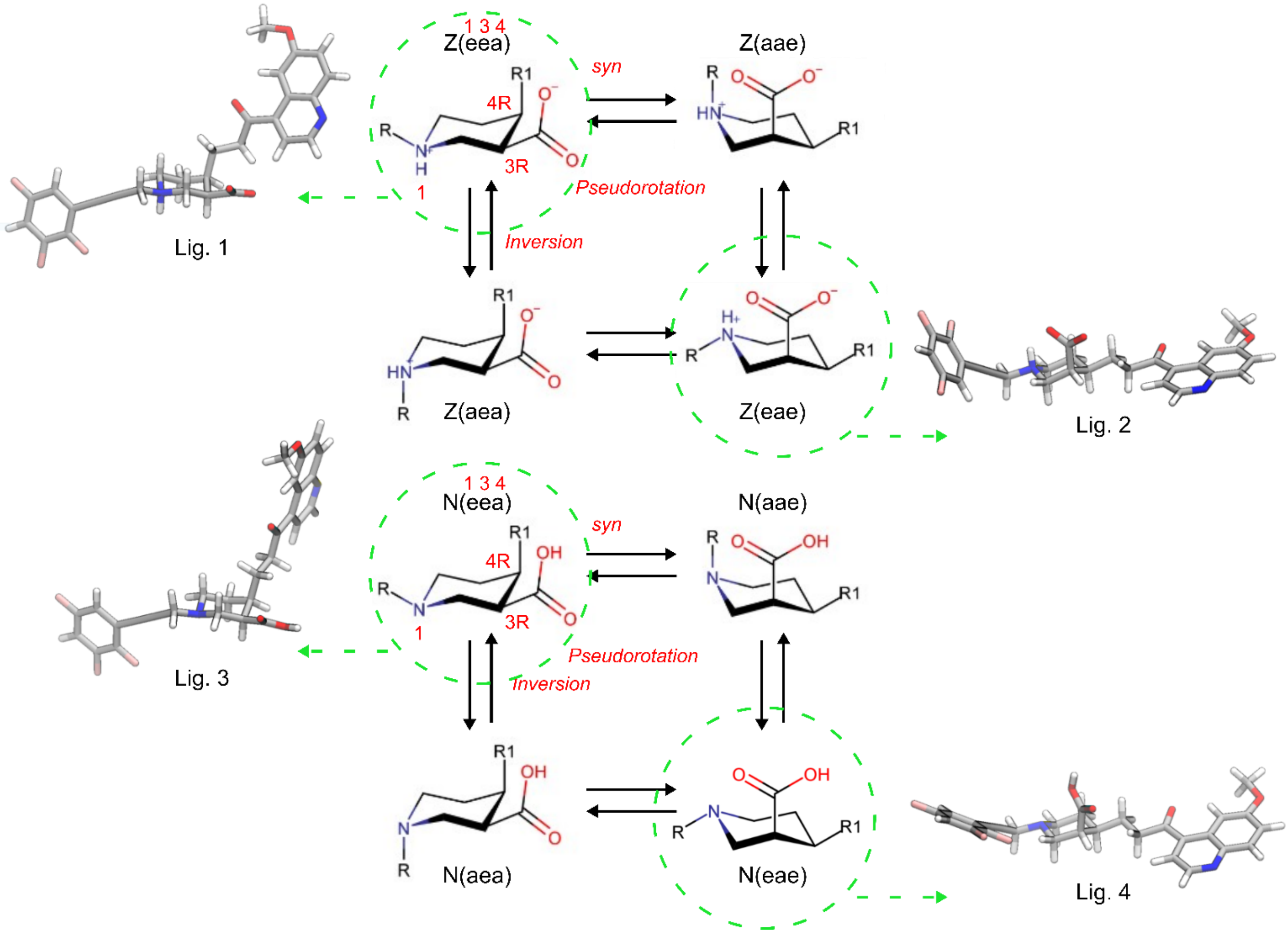

Figure S4. *Notation of the conformational states and protonation forms of RPR included in the docking study.*

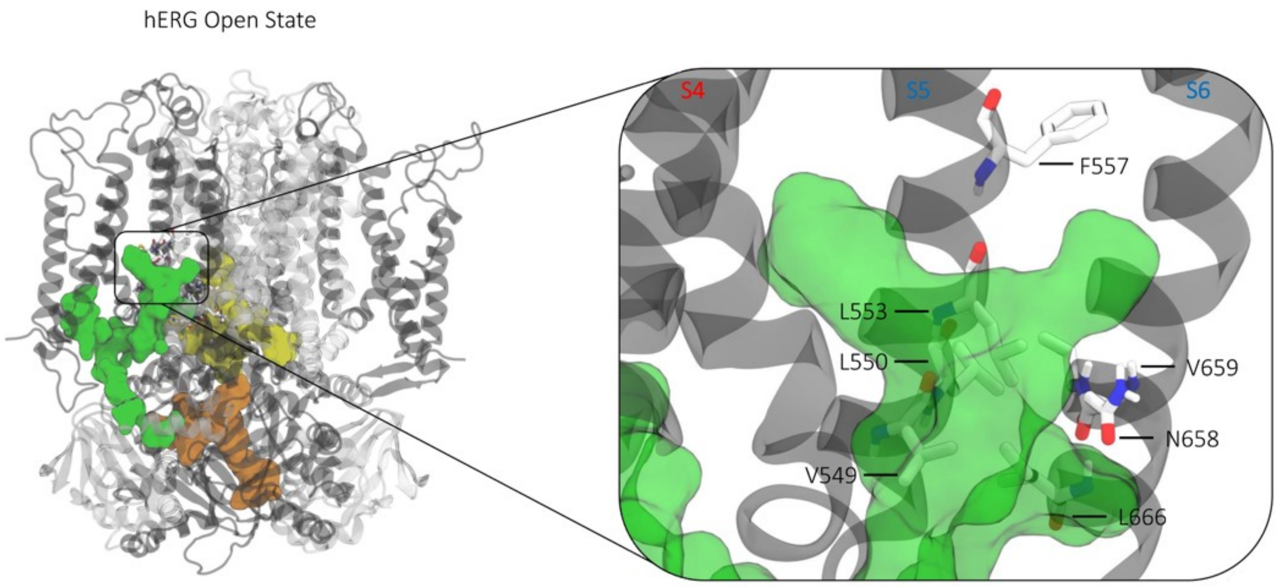

Figure S5. *Three top-ranked pockets in terms of enclosed volume obtained for the open state of the channel #3. Highlighted residues are those identified by Perry et al, to influence the effect of RPR on the hERG gating.*

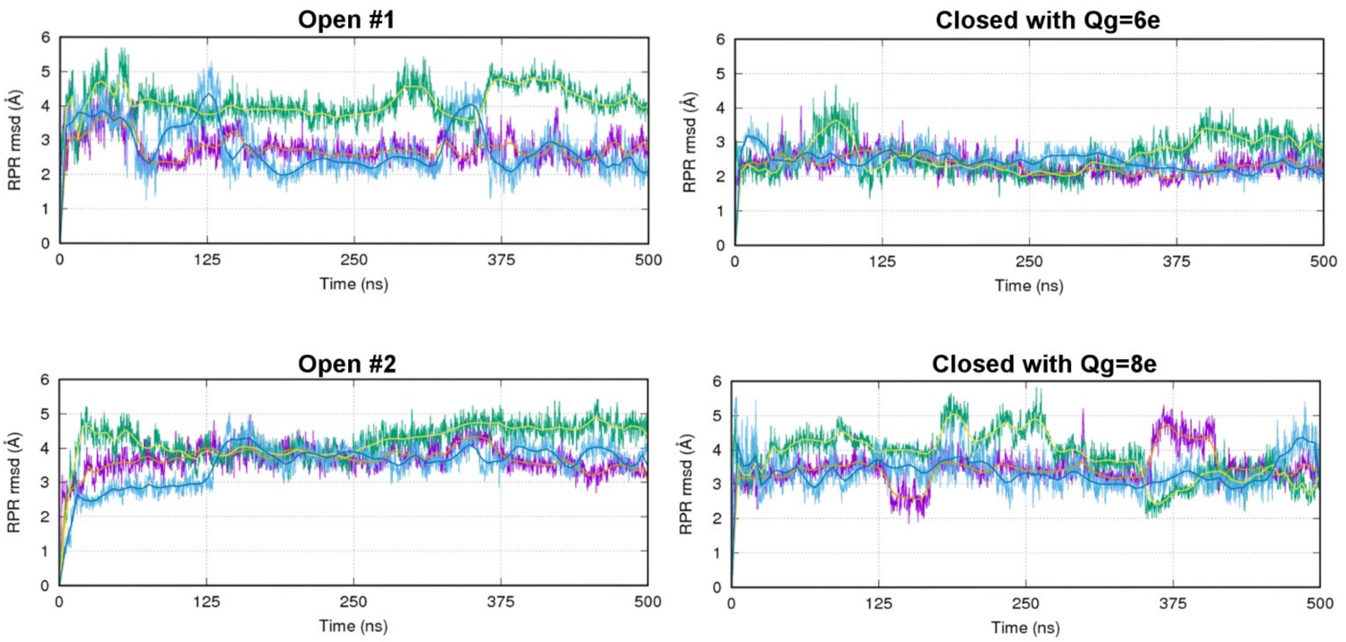

Figure S6. *RMSD profiles of the ligand inside the predicted binding sites during the MD simulations.*
